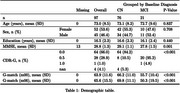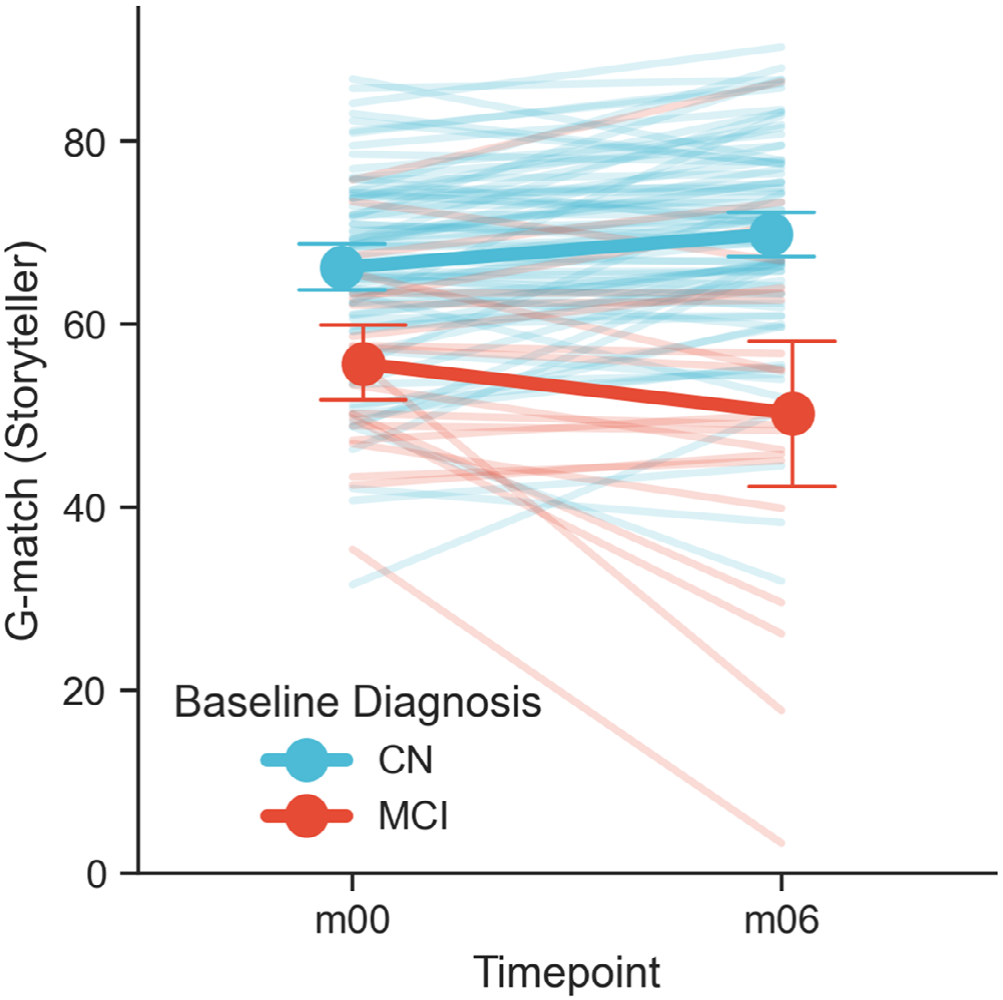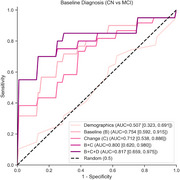# Follow‐up remote cognitive testing improves detection of Mild Cognitive Impairment: initial results from Storyteller in ADNI4

**DOI:** 10.1002/alz70857_099538

**Published:** 2025-12-24

**Authors:** Emil Fristed, Jack Weston, Melanie J. Miller, Michael S. W. Weiner, Rachel L. Nosheny, Alzheimer's Disease Neuroimaging Initiative (ADNI)

**Affiliations:** ^1^ Novoic, London, United Kingdom; ^2^ Northern California Institute for Research and Education (NCIRE), San Francisco, CA, USA; ^3^ University of California, San Francisco, San Francisco, CA, USA; ^4^ Laboratory of Neuro Imaging (LONI), University of Southern California, Los Angeles, CA, USA

## Abstract

**Background:**

Traditional cognitive testing requires trained staff, in‐person visits, and suffers from between‐visit variability, contributing to underdiagnosis of Mild Cognitive Impairment (MCI). Storyteller, a self‐administered remote story‐recall task, enables low‐burden repeat assessments in ADNI4. We examined whether its longitudinal trajectories improve discrimination between MCI and Cognitively Normal (CN) participants.

**Methods:**

ADNI4 participants with baseline clinical diagnosis of CN (*n* = 76) or MCI (*n* = 21) who completed Storyteller at baseline and 6 months were included. Mixed linear regression was used to examine effects of demographic variables (age, sex, education level), baseline diagnosis, and time on Storyteller G‐match scores. Logistic regression models with 10‐fold cross‐validation compared classification performance of baseline G‐match, change in G‐match over a 6‐month period, and both variables together, using Receiver Operating Characteristic (ROC) curve Area Under the Curve (AUC) and 95% confidence intervals.

**Results:**

Age (*p* = 0.837), sex (*p* = 0.708), and education (*p* = 0.440) did not differ between groups, but the MCI group had lower MMSE (*p* = 0.001), G‐match at baseline (*p* <0.001) and 6 months (*p* <0.001); 8 (10.5%) CN participants had CDR‐G 0.5 (Table 1). The mixed model showed significant effects of sex (male: −6.133±2.253, *p* = 0.006), education years (1.221±0.471, *p* = 0.010), baseline MCI (−9.437±2.566, *p* <0.001), timepoint (6 months: 3.622±0.992, *p* <0.001), and MCI*timepoint interaction (MCI declined more: −8.997±2.13, *p* <0.001) (Figure 1). For MCI classification, demographic baseline of sex and education level had an AUC of 0.507 [0.323‐0.691] (*p* = 0.932), while baseline G‐match AUC=0.754 [0.592‐0.915] (*p* = 0.006) and 6‐month change AUC=0.712 [0.538, 0.886] (*p* = 0.022) each outperformed random; combining both improved AUC to 0.800 [0.620‐0.980] (*p* = 0.004), and further including demographics had an AUC of 0.817 [0.659‐0.975] (*p* = 0.001) (Figure 2).

**Conclusion:**

Storyteller detected greater decline in MCI than CN, improving classification when longitudinal measures were included. Remote, self‐administered follow‐up provided additional diagnostic power after only six months.